# Epidemiology and economic burden of herpes zoster and post-herpetic neuralgia in Italy: A retrospective, population-based study

**DOI:** 10.1186/1471-2334-10-230

**Published:** 2010-08-03

**Authors:** Leonardo Emberti Gialloreti, Monica Merito, Patrizio Pezzotti, Luigi Naldi, Antonio Gatti, Maud Beillat, Laurence Serradell, Rafaelle di Marzo, Antonio Volpi

**Affiliations:** 1 Dipartimento di Sanità Pubblica, Università di Roma Tor Vergata, Via Montpellier 1, 00133, Roma, Italy; 2 Informa srl, via del Commercio 36, 00154, Roma, Italy; 3 Centro Studi GISED, Ospedali Riuniti, 24128, Bergamo, Italy; 4 Università degli Studi di Roma Tor Vergata, Fondazione Policlinico Tor Vergata Viale Oxford n° 81, 00133 Roma, Italia; 5 Sanofi Pasteur MSD, 8, rue Jonas Salk, 69367, Lyon, France; 6 Sanofi Pasteur MSD, Via degli Aldobrandeschi, 1500163 Rome, Italy

## Abstract

**Background:**

Data on the epidemiology and cost of herpes zoster (HZ) and post-herpetic neuralgia (PHN) in Italy are limited. This retrospective, population-based study was designed to determine the incidence of HZ and the proportion developing PHN in Italy and the associated medical resource utilisation and costs. It focused primarily on immunocompetent patients aged ≥50 years who would be eligible for preventive vaccination.

**Method:**

Data were extracted from a primary-care database and national hospital-discharge records covering four major regions in Italy for 2003-2005. Cases of HZ and PHN (1 and 3 months' duration; PHN1 and PHN3) were identified by ICD9-CM codes and, additionally for PHN, prescription of neuropathic pain medication.

**Results:**

Over 3 years, 5675 incident cases of HZ were documented in adults, of which 3620 occurred in immunocompetent patients aged ≥50 years (incidence of 6.31 per 1000 person-years [95% CI: 6.01-6.62]). Of the immunocompetent patients aged ≥50 years with HZ, 9.4% (95% CI: 8.2-10.7) and 7.2% (95% CI: 6.2-8.2) developed PHN1 and PHN3, respectively. Increasing age, female sex, and being immunologically compromised conferred increased risk for both HZ and PHN. Overall, about 1.3% of HZ and almost 2% of PHN cases required inpatient care, with 16.9% of all HZ-related hospitalisations due specifically to PHN. In patients aged ≥50 years, mean stay was 7.8 ± 5.4 days for HZ and 10.2 ± 8.6 days for PHN, and direct costs associated with inpatient care were more than 20 times outpatient costs per HZ case (mean ± SD: €2592 ± €1313 vs. €122.68 ± €97.51) and over 5 times more per episode of PHN (mean ± SD: €2806 ± €2641 vs. €446.10 ± €442.97). Total annual costs were €41.2 million, of which €28.2 million were direct costs and €13.0 million indirect costs.

**Conclusions:**

This study, the largest to date on the epidemiology and economic impact of HZ and PHN in Italy, confirms the considerable disease and economic burden posed by HZ. As HZ and PHN disproportionately affect the elderly, without intervention this problem is likely to grow as the proportion of elderly in the Italian population continues to increase.

## Background

Herpes zoster (HZ), caused by reactivation of latent varicella zoster virus present in the dorsal root ganglia, is an acute disease characterised by a vesicular rash. The lifetime risk of developing HZ is about 20-30% [1-3]. However, the risk rises markedly with age, approximately doubling for each decade after 50 years of age [4]. This age-related increase in HZ, which can occur decades after primary varicella zoster infection, is due to a varicella zoster virus-specific decline in cell-mediated immunity with increasing age [5].

While pain associated with the acute infection tends to resolve with the rash, post-herpetic neuralgia (PHN), an intractable pain in the dermatome affected by HZ, remains a common complication. Precise definitions of PHN vary, but it is estimated to affect 10-20% of all patients with HZ aged >50 years [6-8] and up to 30% of those aged ≥80 years [6]. Pain associated with acute HZ infection and more especially PHN causes distress and adversely impacts patients' quality of life [9-14]. Moreover, the management of HZ and particularly PHN, the most common complication in the elderly, is often suboptimal [15,16].

After Japan, Italy has the highest proportion of elderly people in its population [17], and yet data on the epidemiology of HZ and PHN in Italy and the associated costs are limited. An observational study carried out a decade ago in Italy noted an incidence of 4.14 per 1000 population (aged ≥15 years) per year. In that study, over 75% of all HZ cases occurred in those aged ≥50 years, with about 50% occurring in the ≥65-year age group [18]. A more recent prospective Italian study found an annual incidence of 1.59 per 1000 population (aged ≥15 years), and calculated a mean cost for each HZ case treated in the outpatient setting of €360.60 and for each hospitalised case of €4082.59 [19].

The present study was designed to provide additional data on the incidence of HZ and to determine the proportion of HZ cases developing PHN in Italy. The study also sought to determine the associated medical resource utilisation and costs, and to estimate the total economic burden of HZ and PHN in Italy. Both parts of the study focused on the immunocompetent population aged ≥50 years, who would be eligible to receive a preventive vaccine that will be available soon in the European Union.

## Methods

### Study design

This was a retrospective, population-based study in which epidemiological and economic data were estimated in adult men and women, primarily aged ≥50 years, from a large primary-care database and national hospital-discharge records.

### Data sources

Primary-care data were obtained from the Health Search Database (HSD) of the Società Italiana Medici Generici (SIMG) for the period 2003-2005. Established in 1998, and with the input of general practitioners (GPs) who accept to participate on a voluntary basis, the SIMG database contains demographic data, medical diagnoses (coded according to the International Classification of Diseases, 9^th ^Revision, Clinical Modification [ICD9-CM]), drug prescriptions (coded according to the Anatomical Therapeutic Chemical [ATC] classification system), hospital referrals and diagnostic investigations for over 1 million patients [20,21]. This database has been set up by SIMG with the primary aim of carrying out observational studies on incidence and prevalence, as well as studies on drug safety and prescriptions. Data are recorded in the HSD with the consent of the patient, lawfully stored, managed according to privacy rules and can be used for scientific studies without any further authorization from an ethics committee.

The HSD, at the beginning of 2006, contained information from over 600 GPs, who were working in the national public health system, and were from the three major Italian geographical areas (Northern, Central, Southern Italy). The present study included a sample of 342 of these GPs who had contributed medical records to the HSD between 2003-2005. All physicians met standard quality criteria for the HSD [21]. Their geographic distribution mirrored the total population of these three geographical areas (152 from Northern Italy, 64 from Central Italy, and 126 from Southern Italy). The 342 physicians participating in this study represented around 0.8% of the total number of Italian GPs, covering approximately 450,000 patients, of which about 200,000 were aged ≥50 years.

Each participating GP underwent formal training for data entry and used standard software to record data. A unique patient code links demographic and prescription information, clinical events and diagnoses, hospital admission, and cause of death. Data were subject to a range of quality checks.

The primary-care data were supplemented with data from hospital-discharge registries for the period 2003-2005 to determine hospitalisations due to HZ and PHN. Established in 1995 and covering all regions of Italy, the registries use a standard discharge form (Scheda di Dismissione Ospedaliera; SDO) to document patient demographic data, admission and discharge dates, discharge status, diagnostic/therapeutic procedures, discharge primary and secondary diagnoses (coded according to ICD9-CM), and in-hospital transfers. The SDO is the main source of information about hospital admissions in Italy, and all hospital discharges must also be registered, since SDO data contribute to the determination of the appropriate DRG and computation of the reimbursement fee for each hospitalization. Analogous to the HSD, the SDO data are also recorded with the consent of the patient, and can be used as aggregated data for scientific studies without further authorizations.

For the purpose of this study, registries of a representative sample of four regions (Veneto, Toscana, Lazio and Campania), which account for about 30% of the Italian population, were analysed. These regions were chosen as they mirror the three main Italian geographical areas (Northern, Central, and Southern Italy).

### Case definitions used to identify HZ cases in the databases

The overall population of patients included in the analyses of primary-care data were those diagnosed with HZ in years 2003-2005 as defined by ICD9-CM codes (Table 1). To identify the immunocompetent subpopulation, we excluded patients with HZ who had received immunosuppressive or immunomodulating drugs for a period of more than 3 months, and those who had been given the following ICD9-CM codes indicative of immunodeficiency; 042 (human immunodeficiency virus), 140-171, 174-208, and 235-239 (malignancies), 279 (congenital immunodeficiency) and V42 (organ or tissue replaced by transplant). The same case definition was used to identify HZ cases in the hospital-discharge registries.

**Table 1:** International Classification of Diseases (ICD9-CM) Codes for Herpes Zoster and Related Complications

Code Number	Medical Term		
053			Herpes zoster (includes shingles zona)
	053.0		Herpes zoster with meningitis
	053.1		Herpes zoster with other nervous system complications
		053.10	Herpes zoster with unspecified nervous system complication
		053.11	Geniculate herpes zoster/Herpetic geniculate ganglionitis
		053.12	Post-herpetic trigeminal neuralgia
		053.13	Post-herpetic polyneuropathy
		053.19	Other
	053.2		Herpes zoster with ophthalmic complications
		053.20	Herpes zoster dermatitis of eyelid/herpes zoster ophthalmicus
		053.21	Herpes zoster keratoconjunctivitis
		053.22	Herpes zoster iridocyclitis
		053.29	Other
	053.7		Herpes zoster with other specified complications
		053.71	Otitis externa due to herpes zoster
		053.79	Other
	053.8		Herpes zoster with unspecified complication
	053.9		Herpes zoster without mention of complication (also known as herpes zoster NOS)

NOS = not otherwise specified

### Case definitions used to identify PHN cases in the databases

To capture PHN cases seen by a GP in the SIMG database who may not have been recoded from HZ to PHN, patients were included if they had an ICD9-CM code for PHN (Table 1), or an ICD9-CM code for HZ and a prescription for any drug commonly prescribed for PHN, such as opioids, antiepileptics, tricyclic antidepressants (TCAs) or local anaesthetics (Table 2). Because there is no international consensus on the duration of pain in PHN, two definitions of PHN were used for cases identified in the SIMG database. PHN at 1 month (PHN1) or PHN at 3 months (PHN3) included all HZ cases that had been treated at least once with one or more of the aforementioned drugs during the period 1-12 months or 3-12 months after HZ diagnosis, respectively, or had an ICD9-CM PHN code recorded within the same time frame.

**Table 2:** Anatomical Therapeutic Chemical (ATC) Codes Used to Identify PHN Cases in the SIMG Database

ATC Code	Drug Class	Drugs
N02A	Opioids	Tramadol
N03	Antiepileptics	Phenytoin
		Carbamazepine
		Gabapentin
		Pregabalin
N06A	Antidepressants	Amitriptyline
		Nortriptyline
		Imipramine
		Desipramine
N01B	Local	Capsaicin cream
	anaesthetics	Lidocaine

PHN cases were identified in the SDO databases as those with an ICD9-CM code for PHN (053.12, 053.13, or 053.19).

### Statistical analyses

#### Estimates of HZ and proportion of patients with HZ developing PHN

As short-term recurrences of HZ are very uncommon among immunocompetent patients, all cases were assumed to be incident cases. Incidence of HZ was calculated as the number of cases in relation to the population at risk at the beginning of each calendar year (2003, 2004, 2005), defined as the total patient population registered with the 342 primary-care practices. The proportion of patients with HZ who developed PHN (PHN1 or PHN3) was calculated. Estimates were obtained for immunocompetent patients aged ≥50 years (primary analyses), for the whole adult population, and in relation to sex and immune status and by age group. The incidence in immunocompetent patients aged ≥50 years (or incidence in the whole adult population) was extrapolated, by applying appropriate multiplication factors, to provide estimates of the annual number of cases of HZ or PHN for the total immunocompetent population aged ≥50 years (or total adult population) in Italy. Population sizes were obtained from the Italian National Institute of Statistics (ISTAT) website [22].

Rates of hospitalisation for HZ and PHN (immunocompetent patients only) were calculated as the number of hospitalisations for HZ and PHN, extracted from the SDOs, in relation to the population at risk. The population at risk constituted the resident population in each of the four regions, with figures obtained for the period 2003-2005 from the ISTAT website [22].

Univariate and multiple Poisson models, with age group, sex, immune status, region and calendar year as covariates, were used to identify factors associated with an increased risk of acquiring HZ and PHN. As assumed for a poisson model our response variable is a count variable [i.e., 0 no HZ (or 0 no PHN), 1 HZ (or PHN)]. For each year of the study period, each subject was considered exposed for the entire year. Crude and adjusted incidence rate ratios were estimated from these models given that the poisson regression coefficients are the logarithm of the rate ratio. Given that the study design was a two-stage design because data from primary care were clustered by GPs, and those from hospitalisations were clustered by region, the reported 95% confidences intervals were calculated by taking into account the fact that people were clustered within GP practices (for the primary care data) and within region (for the hospitalisation data) [23]. The analyses were performed using Stata software (version 9.0).

#### Estimation of medical resource use and direct and indirect costs associated with HZ and PHN in immunocompetent patients aged ≥50 years

Medical resource consumption for outpatient care in immunocompetent patients aged ≥50 years was extracted from the SIMG database for the period 2003-2005. The follow-up period for cases of HZ was 1 month from diagnosis, while cases of PHN were followed for at least 6 months from initial diagnosis. Estimated unit costs for outpatient visits, diagnostic tests and procedures were based on the average actual cost of each visit/test/procedure across all individual records in the SIMG database in 2005. Costs of medication for HZ and PHN were calculated based on drug prices in the 2005 edition of the Prontuario Farmaceutico Nazionale (PFN) [24]. The amount of resource use and the unit costs were used to calculate the costs of outpatient care per episode of HZ and PHN.

Medical resource consumption and costs for inpatient admissions and hospital day care between 2003 and 2005 in immunocompetent patients aged ≥50 years were obtained from the hospital-discharge registries. Hospital costs were expressed in 2005 prices using the general price index before aggregating all direct costs.

The overall direct costs of HZ and PHN were calculated by multiplying the mean cost of each case with the expected total number of cases in the Italian population.

Indirect costs associated with HZ and PHN were based on likely productivity losses for individuals aged 50-64 years. A focus group of experts, including a neurologist, a geriatrist, a dermatologist and a pain specialist, led by an external moderator, reached a consensus on the number of working days lost due to HZ or PHN. Average daily wage data were taken from Consiglio Nazionale dell'Economia e del Lavoro (CNEL) labour cost statistics [25] and workforce participation rates from ISTAT data [22]. Indirect costs were expressed in 2005 prices using the general price index.

#### Estimates of the economic burden of HZ and PHN in the immunocompetent population aged ≥50 years

Total cost per episode of HZ and PHN was estimated from both the third-party payer and societal perspective. The economic burden of HZ and PHN (calculation restricted to PHN1, as PHN1 and PHN3 were only distinguishable in the outpatient setting) was calculated as the sum of the estimated total outpatient costs, total hospitalisation costs, and total indirect costs (productivity loss) described above.

## Results

### Epidemiological analysis

#### HZ incidence

**Figure 1:**
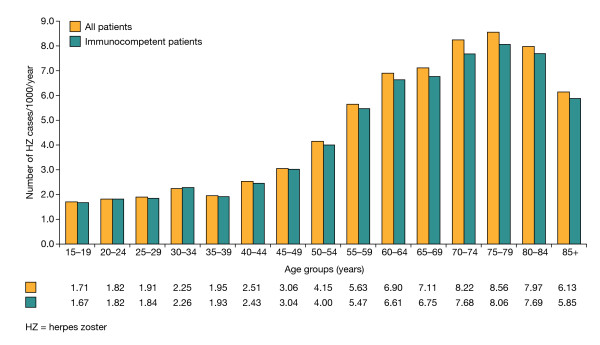
**Incidence of HZ in the general adult (≥15 years) and immunocompetent adult populations in Italy**.

In total, 5675 incident cases of HZ were documented over the 3-year study period, with 1843, 1898 and 1934 cases recorded in 2003, 2004 and 2005, respectively. Patients aged ≥50 years accounted for 4119 (72.6%) incident cases, of which 3620 (87.9%) occurred in immunocompetent patients. Among men and women aged ≥50 years, this corresponded to an incidence of 6.65 per 1000 person-years (95% CI: 6.35-6.97), or 6.31 per 1000 person-years (95% CI: 6.01-6.62) in the immunocompetent population. In comparison, the incidence was 4.31 per 1000 person-years (95% CI: 4.11-4.52) for the adult population as a whole, or 4.07 per 1000 person-years (95% CI: 3.88-4.27) for the immunocompetent adult population. Incidence increased markedly with age, with the peak occurring in those aged 75-79 years (Figure 1). HZ was more common among women than men in the total population, with an incidence of 4.75 (95% CI: 4.47-5.03) compared with 3.82 (95% CI: 3.62-4.03) per 1000 person-years, respectively.

In the multivariate analysis, classical risk factors (female sex, age ≥55 years, and being immunologically compromised) were found to be independently associated with an increased risk of acquiring HZ infection.

#### Projected number of HZ cases per year

With over 22 million people aged ≥50 years living in Italy in 2005 [22], 139,435 new cases of HZ can be expected to occur annually in immunocompetent people aged ≥50 years. A total of 157,100 cases would be expected in all people aged ≥50 years, which represents 72.6% of the 216,391 cases expected for the adult population as a whole. Italian primary-care physicians, who have an average patient registry of 1283, can expect to see between 5 and 6 new cases of HZ per year.

#### Proportion of patients with HZ who developed PHN

**Figure 2:**
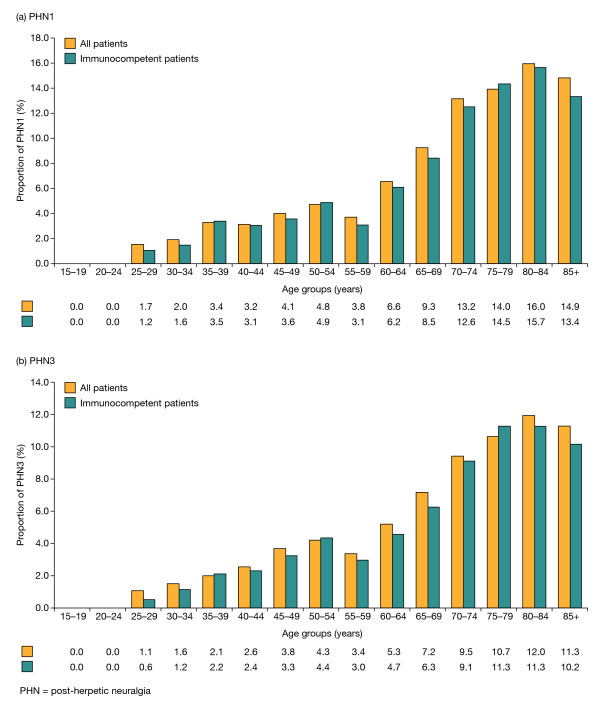
**Proportion of PHN in the general adult (≥15 years) and immunocompetent adult populations in Italy**.

In total, 452 cases of PHN1 were documented in the adult population over the 3-year study period, of which 350 (77.4%) cases also met criteria for PHN3. Thus, 8.0% (452/5675) (95% CI: 7.0-8.9) of the adult population with HZ experienced PHN lasting at least 1 month and 6.2% (350/5675) (95% CI: 5.3-7.0) experienced PHN for at least 3 months. The proportion of patients with HZ who developed PHN was highest in those aged ≥50 years (Figure 2), with these older patients accounting for 91.2% (412/452) of all cases of PHN1 and 90.9% (318/350) of all cases of PHN3. The PHN1 proportion and PHN3 proportion were similar for all HZ patients aged ≥50 years (10.0% [412/4119] [95% CI: 8.8-11.2] and 7.7% [318/4119] [95% CI: 6.6-8.8], respectively) and the immunocompetent HZ patients aged ≥50 years (9.4% [342/3620] [95% CI: 8.2-10.7] and 7.2% [261/3620] [95% CI: 6.2-8.2], respectively).

PHN was more common in women than in men with HZ. In the adult population as a whole, the proportion of women vs. men with HZ who developed PHN was 8.9% (294/3296) (95% CI: 7.7-10.2) vs. 6.6% (158/2379) (95% CI: 5.5-7.7) for PHN1 and 6.9% (228/3296) (95% CI: 5.8-8.0) vs. 5.1% (122/2379) (95% CI: 4.1-6.1) for PHN3. Over 65% of cases of both PHN1 and PHN3 in primary care were documented in female patients.

In the multivariate analysis, classical risk factors (female sex, increasing age [>60 years], and being immunologically compromised) were found to be independently associated with an increased risk of developing PHN.

#### Hospitalisation for HZ and PHN

**Figure 3:**
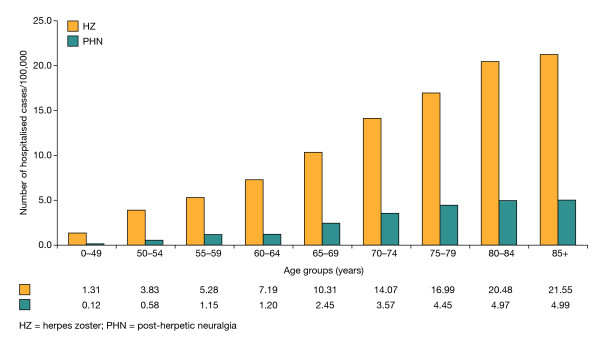
**Age-related incidence of hospitalisation for HZ and PHN in immunocompetent adults in Italy**.

Over the 3-year study period, the average incidences of hospitalisation for primary diagnoses of HZ and PHN were 10.34 and 1.89 per 100,000 person-years, respectively, in immunocompetent patients aged ≥50 years. The total incidence rose to 20.31 per 100,000 person-years (17.45 for HZ and 2.86 for PHN) in this population when both primary and secondary diagnoses on SDOs were combined. Corresponding figures for the adult immunocompetent population as a whole were 5.55 per 100,000 person-years for a primary diagnosis (4.61 for HZ and 0.94 for PHN) and 9.60 per 100,000 person-years for primary and secondary diagnoses combined (7.94 for HZ and 1.66 for PHN). Within the older population, rates of hospitalisation increased progressively for each decade up to 85 years (Figure 3). Based on these figures, HZ or PHN as a primary diagnosis is responsible for 2829 hospitalisations annually in Italy among the immunocompetent adult population, of which 2611 (92.3%) admissions are in those aged ≥50 years. When primary and secondary diagnoses are combined, about 1.3% of HZ cases in Italy result in hospitalisation, rising to almost 2% where patients develop PHN. Overall, 16.9% of hospitalisations related to HZ were due specifically to PHN.

### Economic analysis

#### Outpatient resource consumption

For immunocompetent patients aged ≥50 years, the mean number of primary-care consultations for each case of HZ was 1.9. In total, 17.7% (640/3620) of cases were referred to a specialist (primarily ophthalmologists, dermatologists or neurologists). The mean number of specialist visits was 0.2 for each case of HZ, increasing slightly with age. Antivirals were used in 78.4% of cases, while 12.2% were treated with anticonvulsants, 5.1% with opioid analgesics, 3.2% with non-opioid analgesics and 3.0% with TCAs. Overall, 28.0% (1012/3620) of cases underwent further diagnostic or laboratory examinations, with the mean number of procedures and examinations being 2.6 for each case of HZ, increasing with age (age class range 2.3-2.9).

For immunocompetent patients aged ≥50 years, the mean number of primary-care consultations for each case of PHN1 and PHN3 was 11.9 and 12.0, respectively. In total, 74.1% (206/278) and 73.7% (154/209) of PHN1 and PHN3 cases were referred to a specialist (primarily ophthalmologists, dermatologists or neurologists), with a mean number of specialist visits of 3.5 for each case of PHN1 (age class range 2.2-4.3) and 3.9 for each case of PHN3 (age class range 2.5-4.7). Anticonvulsants were prescribed in 51.4% and 46.4% of PHN1 and PHN3 cases, respectively. Corresponding figures for opioid analgesics were 27.0% and 26.3% and for TCAs were 15.1% and 13.4%, respectively. Drug utilisation was higher overall for cases of PHN3.

#### Direct costs - outpatient care

For immunocompetent patients aged ≥50 years, the mean outpatient cost (±SD) for each case of HZ was €122.68 ± €97.51 (at 2005 prices), of which 83.1% was due to the cost of medications. Diagnostic radiology or ophthalmological and otological examinations (6.8%), laboratory investigations (6.6%) and specialist visits (3.6%) accounted for the remaining costs.

For immunocompetent patients aged ≥50 years, the mean outpatient cost (±SD) for each case of PHN1 was €446.10 ± €442.97 and for each case of PHN3 was €416.65 ± €406.30 (at 2005 prices), of which laboratory investigations and investigative procedures accounted for 58.1% and 63.4% of total costs for PHN1 and PHN3, respectively. Medications accounted for about a quarter of total outpatient costs, at 27.7% and 21.5% for PHN1 and PHN3, respectively. Specialist referral was sought in 74.1% (206/278) of PHN1 cases and 73.7% (154/209) of PHN3 cases; these visits accounted for the remaining 14.2% and 15.1%, respectively, of total outpatient costs.

#### Hospital resource consumption

Of the total hospitalisations for HZ, 92.3% were related to major skin disorders, eye disorders, infections of the nervous system (excluding meningitis) and viral illnesses. For immunocompetent patients aged ≥50 years, the mean (±SD) inpatient stay for HZ was 7.8 ± 5.4 days, which increased with age (6.5 ± 5.5 days for 50-54-year-olds vs. 8.9 ± 5.6 days for those aged >85 years). Of the total hospitalisations for patients with PHN, 92.6% were related to cranial and peripheral nerve disorders and infections of the nervous system (excluding meningitis). For immunocompetent patients aged ≥50 years, the mean (±SD) inpatient stay for PHN was 10.2 ± 8.6 days and showed no age-related trend.

#### Direct costs - inpatient care

For immunocompetent patients aged ≥50 years who were hospitalised, the mean cost (±SD) per patient of inpatient care in the period 2003-2005 was €2592 ± €1313 for HZ and €2806 ± €2641 for PHN.

#### Total direct costs per case

The mean direct medical cost per HZ episode in Italy was calculated as €166, which comprised outpatient costs of €123 per episode, to which €43 was added as the mean cost of hospitalisation per HZ episode, computed as the cost of hospitalisation spread over the total number of expected cases (Table 3). The corresponding mean direct medical cost of PHN (calculation restricted to PHN1, as PHN1 and PHN3 were only distinguishable in the outpatient setting) was calculated as €560 per episode (Table 3).

**Table 3:** Costs Associated with HZ and PHN in Immunocompetent Patients Aged ≥50 years (at 2005 prices)

	HZ	PHN	HZ plus PHN		
Number of expected cases in Italian population per year	129,435	12,228 (PHN1 only*)			
	**Mean €**	**Total €m**	**Mean €**	**Total €m**	**Total €m**
Direct outpatient costs	123	15.9	446	5.3	21.2
Direct hospital costs	43	5.6	114	1.4	7.0
Total direct costs	166	21.5	560	6.7	28.2
Indirect costs	556	12.2	795	0.8	13.0

**Total costs**		**33.7**		**7.5**	**41.2**

HZ = herpes zoster; PHN = post-herpetic neuralgia

* Restricted to PHN1 because PHN1 and PHN3 were only distinguishable in the outpatient setting

#### Indirect costs per case

Mean indirect costs for the immunocompetent population aged ≥50 years were based on the following assumptions: a workforce participation rate of 65.8% for 50-54-year-olds declining to 20.2% for 60-64-year-olds and retirement at 65 years; an average daily wage of €80; and an average loss of 7 and 10 working days for HZ and PHN, respectively. Indirect costs were calculated at €556 and €795 per case of HZ and PHN, respectively (Table 3).

#### Total costs

Based on an estimated 129,435 cases of HZ and 12,228 cases of PHN1 per annum in the immunocompetent population aged ≥50 years, the total direct medical cost of managing HZ infection and PHN in Italy was €28.2 million, of which €21.5 million was associated with the treatment of acute HZ (Table 3). Outpatient costs accounted for 75.2% of the total expenditure. Total indirect costs were calculated as €13.0 million, of which €12.2 million was attributed to acute HZ (Table 3). The total annual economic burden of HZ and associated PHN in the Italian immunocompetent population aged ≥50 years was €41.2 million, with almost a third of the cost attributable to indirect costs associated with lost productivity.

## Discussion

This retrospective, population-based study represents the largest investigation to date of the epidemiology and economic impact of HZ and PHN in Italy. Drawing on a primary-care database of over 450,000 patient records, together with data from national hospital-discharge records covering four major regions in Italy, our study showed that the incidence of HZ in immunocompetent individuals aged ≥50 years (6.31 per 1000 person-years), the primary focus of the study, was comparable with that reported in other studies [8,26], including an earlier Italian study [18]. Furthermore, the incidence reported here was consistent with recently published figures, for the population aged ≥50 years, from the UK (5.23 per 1000 person-years) [6] and Australia (9.7 per 1000 person-years) [7] despite some differences in study design. However, our overall HZ incidence is higher than the one recently published in Italy (4.31 vs. 1.59 per 1000 person-years) [19]. In common with these studies, HZ incidence increased markedly with age up to 80 years. In Italy, most patients with HZ consult their primary-care physician [18] and so our data, retrieved from the SIMG database, can be considered a reliable and robust source for estimates of HZ incidence.

HZ incidence was more common in women than men, a finding supported by several other studies [6,26-28], albeit not all [4,29]. In addition to older age and immune status, female sex was an independent risk factor for HZ, and indeed, for PHN as well. It is known that immunosuppression predisposes patients to increased risk of HZ and its complications [30]. It has been suggested that women may have a different immune response to latent viral infection and that this may account for their increased risk compared with men [27].

PHN, which in our study was identified by ICD9-CM codes or prescriptions for specific neuropathic pain medications, affected 9.4% of immunocompetent patients aged ≥50 years, of whom about 77% continued to have pain 3 months after HZ onset. These rates are consistent with those reported for several other community-based studies [31-33]. However, they are considerably lower than rates reported in clinical trials [34-36], in other various studies [8,26] and, interestingly, in a recent prospective study conducted in Piemonte in Italy [19] and the recent UK General Practice Research Database (UKGPRD) analysis [6], where 17.4% and 19.5% of patients developed PHN1, respectively.

Certain limitations of this study may have resulted in an underestimation of the proportion of PHN. In Italy, primary-care physicians manage most cases of HZ but only about a quarter of related cases of PHN because most cases of PHN are managed by specialists. Thus, this study did not capture cases of PHN that were not seen by a primary-care physician or referred to hospital. This demonstrates that use of the SIMG database to evaluate PHN in Italy has limits as it excludes patients who may have approached specialists directly. In addition, estimating the number of PHN cases retrospectively on the basis of drug prescriptions for pain relief rather than on the basis of pain severity may have led to an underestimation of the proportion of HZ cases that developed PHN because less serious cases which were not treated with drugs and serious cases where patients refused drug treatment may not have been detected. While our estimates of HZ incidence are robust, those for the proportion of patients with HZ who developed PHN are less reliable and are probably underestimates. Indeed, it is arguable that no study has produced reliable estimates of the proportion of HZ cases that develop PHN, and this is exemplified by the variation seen between studies [6,19,26,34,35]. Furthermore, in retrospective studies based on data retrieved from large databases, inaccuracies and, therefore, underestimations due to incompleteness of data cannot be excluded. Prospective studies, with patient follow-up, would provide a more reliable approach to future estimates of the proportion of patients with HZ developing PHN.

Some patients with HZ and PHN require hospital treatment, including inpatient care. Overall, about 1.3% of HZ cases required inpatient care in this study, increasing to almost 2% where only PHN cases were considered. At 9.60 per 100,000 person-years, the incidence of hospitalisations for HZ or PHN was higher in our study than rates reported in other European countries or North America [37,38] despite the exclusion in our study of immunocompromised patients who constitute those at greater risk of complications of HZ. In the recent prospective study in Piemonte, the annual incidence of hospitalisations for HZ was 0.12 per 1000 population [19]. Similar to the Piemonte study, age was an important predictor of likely hospitalisation for HZ or PHN in our study, with rates of hospitalisation increasing progressively for each decade up to 85 years of age, an observation that has also been reported previously [7]. Our data show a striking age-related increase in hospitalisation due to both HZ and PHN whether as a primary or secondary diagnosis.

We also estimated the economic impact of HZ and its complications in terms of direct healthcare costs as well as estimating likely indirect costs. As this study has demonstrated, HZ and PHN, the most common complication of HZ, impose a significant economic burden on the Italian population, with total costs for patients aged ≥50 years exceeding €41 million a year. At €28.2 million, direct medical costs accounted for just over two-thirds of total costs. Indirect costs associated with lost productivity were responsible for almost a third of total costs in the older population and reflect the debilitating nature of HZ and PHN among older people of working age. Although the recent prospective study in Piemonte did not extrapolate data to obtain total direct costs for the Italian population, at €136.06 per observed outpatient case of HZ, €360.60 per case treated in the outpatient setting, and €4082.59 for each hospitalised case (€2499.67 where HZ was the principal diagnosis) direct costs per case were broadly similar to our estimates [19].

Studies have consistently shown that HZ and especially PHN significantly impact patients' quality of daily life [9-14] and, moreover, are difficult to treat. Therapy relies on antiviral drugs to address the underlying cause of HZ and analgesia for pain relief in patients who develop PHN. As this study has shown, medications were responsible for over 80% of all direct outpatient costs, a finding supported by other studies [6,39]. This primary cost determinant may vary widely between countries. In the present study 78% of patients received antivirals, similar to the proportion reported for a recent study in Australia, whereas less than 25% of patients with HZ in The Netherlands receive antiviral therapy [7,40]. If administered within 72 hours of the onset of rash, antivirals have been shown to promote rash healing and reduce the intensity and pain of acute HZ [35,41]. However, a recent Cochrane Review found no evidence for an impact of antiviral therapy on PHN, with their efficacy for prevention of PHN considered modest at best [42].

Previously, it has been shown that patients requiring hospitalisation are likely to have more severe HZ or PHN, and thus, hospital-discharge data provide a measure of more serious cases [2]. In the present study, direct costs associated with inpatient care for an HZ case were more than 20 times the cost for outpatient care. In comparison, direct costs associated with inpatient care for a PHN episode were more than five times the cost for outpatient care. This illustrates the substantial costs associated with the management of more serious cases of HZ and PHN, where patients may require hospital care.

It is difficult to make direct comparisons between the present study and studies carried out in other countries to assess direct and indirect costs associated with HZ and PHN because of differences in healthcare practices and pricing. Nonetheless, as others have concluded, the economic and social costs of managing HZ and its complications represent an important burden on healthcare services and society [43], a view with which we would concur.

Our study had a number of limitations in addition to those already discussed. The higher percentage of home consultations in the very elderly, which may not have been recorded in the SIMG database, may have led to underestimation of incidence in the very old. Hospital-discharge data do not state unequivocally that HZ or PHN was the initial reason for hospitalisation, which may have affected the estimates of disease burden. As GP prescriptions could not be linked to specific PHN diagnostic codes and the drugs may have been prescribed for conditions other than PHN, outpatient costs for PHN may have been overestimated. Indirect costs were based solely on productivity losses due to absence from work and were derived from expert opinion, an approach that may limit the reliability of indirect cost estimates. Experts agreed that HZ patients will usually loose between 7 and 10 working days, while in case of serious pain 10-15 working days are reasonably lost due to PHN. Accordingly, the conservative assumption of 7 and 10 working days lost due to HZ and PHN, respectively, was made in the estimate of indirect costs. However, all focus group specialists stressed the extreme variability of the non-acute phase. Finally, when combining the results from the epidemiological and economic parts of the study it was not possible to obtain a measure of the reliability of estimates of the total cost of HZ and PHN (covariance between the different components of the total variability was unknown).

## Conclusions

In conclusion, this study has demonstrated that HZ and related complications such as PHN impose a considerable disease and economic burden in Italy. While our estimates of HZ incidence in the Italian population can be considered robust, those for the proportion of patients with HZ who develop PHN are likely to be underestimates and would best be assessed in prospective studies. As HZ and PHN disproportionately affect the elderly, without intervention the burden to patients and society is likely to grow as the proportion of elderly in the Italian population continues to increase.

## Competing interests

LN provided consultancy advice to Sanofi Pasteur MSD to assist with developing the study. AG has provided consultancy advice to Sanofi Pasteur MSD. AV has provided consultancy advice to Sanofi Pasteur MSD and Novartis, and has received honoraria for lectures from these companies and from GlaxoSmithKline and Menarini. He has also received honoraria for preparing educational materials for Menarini. LEG, MM and PP are consultants for Informa, Rome, who coordinated this study. MB, LS and RM are employees of Sanofi Pasteur MSD, a provider of a herpes zoster vaccine approved in the European Union.

## Authors' contributions

LEG participated in the study design, analysis of data and drafting of the study report and helped to draft the manuscript. MM and PP participated in the study design and analysis of data. LN, AG and AV validated the protocol, design and final results of the study. MB, LS and RM participated in the study design, coordinated the study and validated the study results and study report. All authors read and approved the final draft manuscript.

## Pre-publication history

The pre-publication history for this paper can be accessed here:

http://www.biomedcentral.com/1471-2334/10/230/prepub
